# Estrogen and progesterone receptor levels in nonneoplastic breast epithelium of breast cancer cases versus benign breast biopsy controls

**DOI:** 10.1186/1471-2407-8-130

**Published:** 2008-05-08

**Authors:** Christy G Woolcott, Sandip K SenGupta, Wedad M Hanna, Kristan J Aronson

**Affiliations:** 1 Cancer Research Center of Hawaii, University of Hawaii, Honolulu, Hawaii, 96813, USA; 2 Department of Pathology and Molecular Medicine, Queen's University, Kingston, Ontario, K7L 3N6, Canada; 3 Department of Pathology, Sunnybrook Health Sciences Centre, Toronto, Ontario, M5S 1B2, Canada; 4 Department of Laboratory Medicine and Pathobiology, University of Toronto, Toronto, Ontario, M5G 1L5, Canada; 5 Department of Community Health and Epidemiology, and Division of Cancer Care and Epidemiology, Cancer Research Institute, Queen's University, Kingston, Ontario, K7L 3N6, Canada

## Abstract

**Background:**

Previous studies and biological mechanisms of carcinogenesis suggest that the steroid receptor content of benign breast epithelium may be related to breast cancer risk. The objective in this study was to compare the levels of estrogen receptor-α (ER) and progesterone receptor (PR) in nonneoplastic breast epithelium between breast cancer cases and biopsy controls.

**Methods:**

Between 1995 and 1997 at two sites (Women's College Hospital in Toronto and Kingston General Hospital), 667 women who were scheduled for diagnostic excisional breast biopsies completed a questionnaire providing personal information and agreed to allow analysis of routinely resected tissue. Histological slides with nonneoplastic epithelium were available for 101 cancer cases and 200 biopsy controls in Toronto and for 105 cancer cases and 119 controls in Kingston. Nonneoplastic epithelium was examined with immunohistochemical assays to determine the percent of epithelial cells staining for ER and PR. Unconditional logistic regression was used to calculate odds ratios (OR) stratified by study site.

**Results:**

The ER content of nonneoplastic tissue was higher in cases than biopsy controls in unadjusted analyses; after adjustment for age, however, a weak association remained in only one of the study sites. After adjustment for age, the PR content of nonneoplastic tissue was slightly lower in breast cancer cases than controls in one study site. Furthermore, this inverse association was confined to women with PR negative breast cancer in comparison to the controls. No interaction between ER and PR content of nonneoplastic tissue was observed in relation to the odds of having breast cancer.

**Conclusion:**

The results of this study are consistent with only a slight indication of increased ER levels in nonneoplastic tissue in breast cancer cases relative to controls. This study contributes to the understanding of breast cancer by examining both ER and PR in nonneoplastic tissue. Limitations remain, however, such as the necessity of using as controls women with benign breast changes, difficulties in selecting the appropriate tissue for analysis, and tissue sampling concurrent to diagnosis.

## Background

Breast cancer risk factors such as early age at menarche, late age at menopause, postmenopausal hormone therapy, and high body mass index are thought to affect breast carcinogenesis by increasing the exposure of the breast to estrogens and other sex hormones [1,2]. Estrogens, particularly in conjunction with progesterone, are mitogenic to breast epithelial cells. The resulting proliferation could increase the probability of mistakes being made in DNA replication and setting these mistakes as mutations. Because the effects of estrogens are mediated by the estrogen receptors (ER), the magnitude of their effects may be determined by the level of ER expressed in the breast. Because breast epithelial cell proliferation is related to both estrogen and progesterone, and progesterone also acts through its own receptor [3], levels of the progesterone receptor (PR) may also be important in breast carcinogenesis. In previous case-control studies, Khan and colleagues observed that the proportion of breast epithelial cells expressing ER was higher in women with breast cancer than benign breast disease controls [4,5].

We previously conducted a hospital-based case-control study of the association between breast cancer risk and breast adipose tissue concentrations of organochlorines such as polychlorinated biphenyls, DDT, and its metabolite DDE [6]. Because both breast cancer cases and controls had a breast biopsy, we took the opportunity to examine nonneoplastic sections for ERα and PR to see if levels of these receptors differed between the breast cancer cases and controls.

## Methods

The methods of the original biopsy case-control study have been reported in detail elsewhere [6] and the methods specific to this study will be described in detail here. The original and extended protocols were approved by the ethics committees at Women's College Hospital and Queen's University/Kingston General Hospital and all subjects provided informed consent.

### Subjects

Subjects were women who had a diagnostic breast biopsy at Women's College Hospital in Toronto or Kingston General Hospital in Kingston between 1995 and 1997. Because the original study depended on obtaining a sample of breast adipose tissue in which to measure organochlorines, biopsy controls were chosen. Before biopsy, the study was presented by the surgeons to all women who did not have previous cancer or breast implants and were under the age of 80. After the biopsy, pathology records were reviewed and all women with *in situ *or invasive breast cancer were cases and all women whose biopsy was negative for malignancy were controls.

Of the 824 eligible women, 667 (80.9%) provided informed consent and completed a questionnaire that collected information about known and suspected risk factors that were considered as potential confounders in the analyses. Mammographic density was also determined in a subset of 359 women whose mammograms were available to us for analysis. The mammograms were digitized and a computer-assisted thresholding program, Cumulus, allowed separation of the breast area from the background and dense area from nondense area. Percent dense area is calculated by dividing the number of pixels representing dense breast tissue by the total number of pixels in the breast [7].

### Immunohistochemistry on nonneoplastic tissue

Routine practice at both hospitals from which subjects were recruited was to store surgical specimens from all diagnostic biopsies. We attempted to locate the stored sections of nonneoplastic tissue of the 667 subjects who completed a questionnaire for this study. Stored sections were located for 561 (84.1%) subjects; because this extension study started two years following the time when the last subject had her biopsy and extensive hospital restructuring occurred at one of the study sites (Toronto) during this time, some slides were unavailable for review. A section of nonneoplastic breast tissue from each subject was chosen by a preliminary review of all of the slides stored. The absence of nonneoplastic tissue or unsatisfactory preparation of slides prevented the analysis of the tissue of 36 subjects. Thus, steroid receptor levels in nonneoplastic tissue were determined for 206 cases and 319 controls.

Tissue sections were first evaluated by hematoxylin and eosin staining to determine if nonneoplastic epithelium was present to continue with steroid receptor studies. The slides chosen had nonneoplastic tissue including a spectrum from atrophic, largely fibrous or fatty breast stroma with only scattered ducts and a minor epithelial component to breast tissues containing large numbers of well formed terminal duct lobular units. In between the two ends of this spectrum was a range of fibrocystic changes, mainly microscopic cysts, lobular unfolding, apocrine metaplasia, and occasional ductal hyperplasia. Rarely the sections of nonneoplastic tissue included a few ducts exhibiting ductal carcinoma *in situ*, but more frequently, the sections showed a fibroadenoma with a small rim of normal breast tissue around it. Histology apparent on the slide was noted.

Steroid receptors were determined using standard immunohistochemical techniques; slides at each site were stained no more than two weeks after being cut at the clinical pathology departments of the respective hospitals due to feasibility. Briefly, 5 μm sections were treated with 3% hydrogen peroxide solution and washed with distilled water. Slides were then microwaved to retrieve the antibody-binding epitope of the antigen and treated with casein solution (0.5%) and drained. The slides were then sequentially treated and incubated with the primary antibody, biotinylated secondary link antibody, and peroxidase-conjugated streptavidin. Slides were developed in stable DAB and counterstained with hematoxylin stain. Differences in the procedures at each hospital include that the staining procedures were automated on a Ventana ES system in Kingston but done manually in Toronto, and the primary antibody against the ER, 6F11, was supplied by Ventana in Kingston and by Novocastra in Toronto, and the primary antibody against PR was 1A6 supplied by Ventana in Kingston and 636 supplied by Dako in Toronto.

The level of receptors was scored as the percent of immunostained epithelial cell nuclei. An experienced pathologist with expertise in breast pathology (SKS) assessed sections at 400× magnification under a light microscope. Although immunohistochemical staining expression was examined across the entire slides including all ducts and lobules, only the nonneoplastic tissue without fibrocystic changes on the slides was scored. The percent of cells staining positively for the receptors was scored within categories: < 1, 1–5, 6–10, 11–33, 34–67, and >67. The pathologist was blind to case-control status.

### Tumor characteristics

Levels of ER and PR within the tumors of the cases had been determined by one or both of immunohistochemistry or enzyme immunoassay in each hospital as part of the routine clinical practice. Using the immunohistochemical assay, tumors were classified as positive if more than 10% of the cells showed nuclear staining for the receptor. Using the enzyme immunoassay, tumors were classified as positive if the concentration of receptor was greater than 10 fmol/mg cytosolic protein. Cases who were positive by at least one assay were considered to have positive receptor status [8]. We obtained the information on ER and PR within the tumors of the cases from the pathology reports.

### Statistical analyses

Because of the differences at each site in the preparation of the nonneoplastic tissue and the immunohistochemical staining, and the differences in the distribution of the ERs by study site that were not explained by case-control status, menopausal status and age, analyses were also conducted separately for each site. Subjects with receptor results were compared to subjects without receptor results with respect to covariates. Among the subjects with exposure measurements, frequencies (categorical variables) and means with standard deviations (continuous variables) of the variables were examined.

The association between ER and PR content of nonneoplastic tissue and breast cancer status was examined by calculating odds ratios (ORs) using multivariable logistic regression; this approach is consistent with other studies and allows direct comparison [4,5]. The level of steroid receptors in nonneoplastic tissue was scored in six categories, but adjacent categories were combined such that at least ten cases and ten controls were in each category. Variables considered as covariates derived from the questionnaire included age (continuous), site (Toronto, Kingston), menopausal status (pre, post), parity (no, yes), lactation duration (continuous), age at first pregnancy (<20, 20–24, 25–29, > = 30, nulliparous), age at last pregnancy (<25, 25–29, 30–34, > = 35, nulliparous), age last breast fed (never, <30, ≥ 30 years), family history (no, breast cancer in first or second degree relative), body mass index (continuous), fat intake (continuous), energy intake (continuous), alcohol intake (<1, ≥ 1 drink per week), current smoking (no, yes), cumulative smoking (continuous), and current hormone use (no, yes). Subjects who reported that their menstrual periods had stopped permanently were classified as postmenopausal. However, six subjects who had a hysterectomy in the absence of bilateral oophorectomy and were under the mean age of menopause of subjects having a natural menopause (49 years) were reclassified as premenopausal. All ORs were adjusted for age, but no other covariate caused the ORs associated with steroid receptor levels to consistently change more than 10% when introduced into the age-adjusted model and thus, were not included in the models. The confounding effect of mammographic density, which had missing values for large numbers of subjects, was examined within the subset of subjects with this measure and was found to be nil.

Sensitivity analyses were done to examine if exclusion of cases with carcinomas *in situ *from the case group, controls with hyperplasia with atypia, subjects who were currently using exogenous hormones, premenopausal women, or subjects with no terminal duct lobular units apparent on the slide had an effect on the conclusions from the main analysis. Polytomous logistic regression was used to estimate the association between ER and PR levels in nonneoplastic tissue and both ER positive breast cancer and ER negative breast cancer relative to the control group. A likelihood ratio test was performed to evaluate the significance of the ER and PR levels in nonneoplastic tissue in a logistic model comparing ER positive cases with ER negative cases. The significance of this test indicated if heterogeneity existed in the associations between ER and PR levels in nonneoplastic tissue and risk of ER-positive and ER-negative breast cancers. Analyses initially were done separately for ER and PR in nonneoplastic tissue, and then they were included in the same model to examine whether they interacted with one another in their association with breast cancer status. For these analyses, PR was dichotomized (<5%, ≥ 5%) and the two uppermost categories for ER were collapsed to ensure sufficient numbers in the cells for the analysis. All analyses were done using SAS (SAS Institute, Cary, NC, USA). A p < 0.05 was used to indicate statistical significance.

## Results

When the 206 cases and 319 controls, the subset of subjects with measurements for ER and PR in nonneoplastic tissue, were compared with the total group of 267 cases and 400 controls who had completed a questionnaire, it was found that more subjects from Toronto (27.1%) had missing receptor results than from Kingston (11.8%); this was observed for both cases and controls. Characteristics of the cases and controls included in this analysis with respect to covariates are shown in Table 1. In both study sites, cases tended to be older than controls, have a slightly higher fat intake, and have smoked more cigarettes. A lower proportion of cases than controls was premenopausal, had never breast fed, reported current use of exogenous hormones, had a family history of breast cancer, and consumed more than one alcoholic beverage per week. A higher percentage of epithelial cells stained positively for ER among cases than controls. Among controls, the ER level in nonneoplastic tissue was associated with age (Spearman correlation, r_s _= 0.49, p < 0.0001). After adjustment for age, fat intake was the only covariate associated with ER level (Spearman correlation, r_s _= 0.14, p = 0.01). Among controls, the PR level in nonneoplastic tissue was not associated with age or any other covariate after age adjustment.

**Table 1:** Characteristics of cases and controls, by study site

	**Toronto**				**Kingston**			
		
**Characteristic**	**Controls (n = 200)**		**Cases (n = 101)**		**Controls (n = 119)**		**Cases (n = 105)**	
Age, mean (SD), y	44.9	(9.6)	54.3	(11.4)	56.1	(11.8)	60.6	(11.4)
Breast feeding, mean (SD), months	5.7	(10.2)	5.3	(9.1)	6.5	(15.9)	6.5	(9.5)
Body mass index^a^, mean (SD), kg/m^2^	23.3	(3.9)	24.3	(4.3)	25.7	(4.5)	25.4	(4.7)
Energy intake^a^, mean (SD), kcal/day	957	(385)	991	(307)	981	(385)	1121	(374)
Fat intake^a^, mean (SD), g/day	33.3	(14.8)	35.1	(12.8)	37.4	(17.9)	43.3	(16.3)
Smoking^a^, mean (SD), pack-years	7.8	(12.9)	9.0	(13.2)	10.9	(15.9)	13.4	(17.1)
Mammographic density^a^, mean (SD), %	41.0	(18.7)	35.8	(16.2)	26.5	(17.6)	26.0	(17.3)
Menopausal status, n (%)	54	(27.0)	59	(58.4)	79	(66.4)	81	(77.1)
Gravid, n (%)	149	(74.5)	78	(77.2)	106	(89.1)	93	(88.6)
Age last breast fed, n (%)								
never	105	(52.5)	45	(44.6)	65	(54. 6)	46	(43.8)
<30	42	(21.0)	24	(23.8)	32	(26.9)	26	(24.8)
≥30	53	(26.5)	32	(31.7)	22	(18.5)	33	(31.4)
Oral contraceptives at questionnaire, n (%)	12	(6.0)	8	(7.9)	1	(0.8)	1	(1.0)
Hormone therapy at questionnaire, n (%)	26	(23.0)	8	(7.9)	27	(22.7)	10	(9.5)
Family history, n (%)	83	(41.5)	35	(34.7)	41	(34.5)	42	(40.0)
Alcohol consumed >1 drink/week^a^, n (%)	103	(52.0)	44	(44.0)	52	(43.7)	37	(35.6)
Current smoking, n (%)	49	(24.5)	18	(17.8)	25	(21.0)	22	(21.0)
Invasive cancer, n (%)	-		88	(87.1)	-		94	(89.5)
Tumor steroid receptor status, n (%)	-				-			
ER+/PR+			55	(54.5)			59	(56.2)
ER+/PR-			10	(9.9)			14	(13.3)
ER-/PR+			7	(6.9)			4	(3.8)
ER-/PR-			24	(23.8)			20	(19.0)
Missing			5	(5.0)			8	(7.6)
Atypical hyperplasia, n (%)	13	(6.5)	-		3	(2.5)	-	
Nonneoplastic cells staining for ER, n (%)								
<1%	63	(31.5)	21	(20.8)	3	(2.5)	2	(1.9)
1–5	66	(33.0)	23	(22.8)	13	(12.4)	4	(3.8)
6–10	43	(21.5)	27	(26.7)	27	(22.7)	24	(22.9)
11–33	17	(8.5)	17	(16.8)	37	(31.1)	23	(21.9)
34–66	9	(4.5)	9	(8.9)	28	(23.5)	25	(23.8)
>66	2	(1.0)	4	(4.0)	11	(9.2)	27	(25.7)
Nonneoplastic cells staining for PR, n (%)								
<1%	20	(10.0)	22	(21.8)	16	(13.4)	16	(15.2)
1–5	70	(35.0)	27	(26.7)	39	(32.8)	36	(34.3)
6–10	62	(31.0)	31	(30.7)	47	(39.5)	31	(29.5)
11–33	27	(13.5)	12	(11.9)	10	(8.4)	12	(11.4)
34–66	12	(6.0)	9	(8.9)	7	(5.9)	8	(7.6)
>66	9	(4.5)	0	(0.0)	0	(0.0)	2	(1.9)

ER, estrogen receptor; PR, progesterone receptor; SD, standard deviation.^a^Information for BMI is missing for 3 subjects, energy, fat and alcohol consumption is missing for 4 subjects, cumulative smoking is missing for 4 subjects, and mammographic density is missing for 166 subjects.

### Association of ER in nonneoplastic tissue and breast cancer status

The crude and age-adjusted ORs for the association between ER levels in nonneoplastic tissue and breast cancer status overall and stratified by study site are shown in Table 2. The crude ORs suggest a positive association between the percentage of cells staining for ER staining in nonneoplastic tissue and breast cancer status in both sites. Age, however, was a strong confounder due to its association with case status (t-test p < 0.005) and with ER levels. When age was taken into account, the ORs were attenuated toward the null value. The OR for the uppermost category of ER (>66% positively staining cells) relative to the lowermost category (<1% cells staining positively) remained elevated (OR = 2.6; 95% confidence interval (CI): 1.1–6.2) but no other OR was significantly different from the null. This was largely due to the influence from the Kingston site. The association between ER and breast cancer status was not different by menopausal status (p-value for interaction = 0.50). Exclusion of cases with carcinoma *in situ *only, controls with atypical ductal hyperplasia, or subjects whose slides contained no TDLUs changed the results for each site very little. When subjects who reported taking exogenous hormones were excluded, the ORs increased slightly for Toronto but were attenuated slightly to the null for Kingston.

**Table 2:** Association between ER in nonneoplastic tissue and breast cancer status, by study site

Site	Controls		Cases			
				
Cells staining for ER	N	(%)	N	(%)	Crude OR (95% CI)	Age-adjusted OR (95% CI)
Both						
<1%	66	(20.7)	23	(11.2)	1.0	1.0
1–5	79	(24.8)	27	(13.1)	1.0 (0.5–1.9)	0.9 (0.5–1.7)
6–10	70	(21.9)	51	(24.8)	2.1 (1.2–3.8)	1.5 (0.8–2.8)
11–33	54	(16.9)	40	(19.4)	2.1 (1.1–4.0)	1.3 (0.7–2.5)
34–66	37	(11.6)	34	(16.5)	2.6 (1.4–5.1)	1.4 (0.7–2.8)
>66	13	(4.1)	31	(15.0)	6.8 (3.1–15.3)	2.6 (1.1–6.2)
Toronto						
<1%	63	(31.5)	21	(20.8)	1.0	1.0
1–5	66	(33.0)	23	(22.8)	1.0 (0.5–2.1)	0.9 (0.5–2.0)
6–10	43	(21.5)	27	(26.7)	1.9 (0.9–3.8)	1.2 (0.6–2.5)
11–33	17	(8.5)	17	(16.8)	3.0 (1.3–6.9)	1.4 (0.6–3.6)
>33	11	(5.5)	13	(12.9)	3.5 (1.4–9.1)	1.4 (0.5–3.9)
Kingston						
≤10%	43	(37.6)	30	(28.6)	1.0	1.0
11–33	37	(31.1)	23	(21.9)	0.9 (0.4–1.8)	0.9 (0.4–1.7)
34–66	28	(23.5)	25	(23.8)	1.3 (0.6–2.6)	1.1 (0.5–2.4)
>66	11	(9.2)	27	(25.7)	3.5 (1.5–8.2)	2.7 (1.1–6.6)

CI, confidence interval; ER, estrogen receptors; OR, odds ratio.

The percentage of nonneoplastic epithelial cells staining positively for ER was not significantly differentially related to ER positive breast cancer status as compared to ER negative breast cancer (p = 0.72), nor was it significantly differentially related to PR positive breast cancer status as compared to PR negative breast cancer (p = 0.44; results not shown). Note, however, that the number of ER negative cancer cases (n = 55) or PR negative cancer cases (n = 68) in our study was small and the power to detect differences was low.

### Association of PR in nonneoplastic tissue and breast cancer status

The crude and age-adjusted ORs for the association between PR levels in nonneoplastic tissue and breast cancer status overall and stratified by study site are shown in Table 3. Among subjects from Toronto, the crude ORs suggest a slightly inverse association between the percentage of PR staining cells in nonneoplastic tissue and breast cancer status; this was not observed in the subjects from Kingston. When age was taken into account, the ORs were only slightly attenuated toward the null value. The associations did not differ significantly by menopausal status (p-values for interaction ≥ 0.06). Exclusion of cases with carcinoma *in situ *only, controls with atypical ductal hyperplasia, subjects whose slides contained no TDLU, or subjects using exogenous hormones would not change the conclusions made from these analyses.

**Table 3:** Association between PR in nonneoplastic tissue and breast cancer status, by study site

Site	Controls		Cases			
				
Cells staining for PR	N	(%)	N	(%)	Crude OR (95% CI)	Age-adjusted OR (95% CI)
Both						
<1%	36	(11.3)	38	(18.5)	1.0	1.0
1–5	109	(34.2)	63	(30.6)	0.5 (0.3–1.0)	0.7 (0.4–1.2)
6–10	109	(34.2)	62	(30.1)	0.5 (0.3–0.9)	0.8 (0.4–1.4)
>10	65	(20.4)	43	(20.9)	0.6 (0.3–1.1)	0.7 (0.4–1.3)
Toronto						
<1%	20	(10.0)	22	(21.8)	1.0	1.0
1–5	70	(35.0)	27	(26.7)	0.4 (0.2–0.7)	0.5 (0.2–1.1)
6–10	62	(31.0)	31	(30.7)	0.5 (0.2–1.0)	0.9 (0.4–2.0)
>10	48	(24.0)	21	(20.8)	0.4 (0.2–0.9)	0.4 (0.2–1.0)
Kingston						
<1%	16	(13.4)	16	(15.2)	1.0	1.0
1–5	39	(32.8)	36	(34.3)	0.9 (0.4–2.1)	1.0 (0.4–2.4)
6–10	47	(39.5)	31	(29.5)	0.7 (0.3–1.5)	0.8 (0.3–1.8)
>10	17	(14.3)	22	(20.9)	1.3 (0.5–3.3)	1.4 (0.5–3.6)

CI, confidence interval; OR, odds ratio; PR, progesterone receptor.

The percentage of nonneoplastic epithelial cells staining positively for PR was not significantly differentially related to ER positive breast cancer status as compared to ER negative breast cancer (p = 0.67). It was, however, significantly differentially related to PR positive breast cancer status as compared to PR negative breast cancer among subjects from Toronto (p = 0.03). Although PR levels in nonneoplastic tissue were not related to PR positive breast cancer status, levels of PR in nonneoplastic tissue above 1% were associated with reduced odds of having PR negative breast cancer status (results not shown). The same trend was seen among subjects from Kingston, but it was not statistically significant (p = 0.19).

### Interaction between ER and PR in nonneoplastic tissue and breast cancer status

Levels of ER and PR in nonneoplastic breast tissue were positively correlated. The partial Spearman correlation (adjusted for age) was r_s _= 0.36 in Toronto controls, r_s _= 0.42 in Kingston controls, r_s _= 0.50 in Toronto cases, and r_s _= 0.65 in Kingston cases. In Kingston, the odds of being a breast cancer case with respect to ER levels was qualitatively higher in women in whom PR levels were higher (Table 4). The interaction, however, was not statistically significant in either site (p > 0.34).

**Table 4:** Joint association between both ER and PR in nonneoplastic tissue and breast cancer status, by study site

Site	Cells staining for PR	
	
Cells staining for ER	≤5%	>5%
Both		
<1%	1.0	1.0
1–5	0.5 (0.2–1.3)	1.8 (0.6–5.9)
6–10	1.4 (0.6–3.3)	1.9 (0.6–5.7)
>10	1.1 (0.5–2.5)	2.6 (0.9–7.6)
	p(interaction) = 0.29	
Toronto		
<1%	1.0	1.0
1–5	0.6 (0.2–1.6)	1.9 (0.6–6.2)
6–10	1.4 (0.5–3.9)	1.3 (0.4–4.5)
>10	1.6 (0.4–5.7)	1.6 (0.4–5.9)
	p(interaction) = 0.34	
Kingston		
≤10%	1.0	1.0
11–33	0.9 (0.3–2.6)	1.2 (0.4–3.9)
>33	1.1 (0.4–2.8)	3.1 (0.9–10.3)
	p(interaction) = 0.69	

ER, estrogen receptor; PR, progesterone receptor.

## Discussion

After age was taken into account, very little association remained between ER levels in nonneoplastic tissue and breast cancer status. The odds of having the highest level of ER were higher in breast cancer cases than biopsy controls only in Kingston, one of our two study sites. Some indication of an inverse association between PR levels in nonneoplastic tissue and breast cancer status was observed among subjects from Toronto, which was largely because of the association of PR levels with PR negative breast cancers; no association between PR levels in nonneoplastic tissue and breast cancer status was observed among subjects from Kingston.

Khan *et al*. [4,5] have found increased ER levels in benign tissue in breast cancer cases than controls even after adjusting for age. In comparison to our unadjusted analyses, they observed an increase in risk for ER levels between 1% and 5% relative to less than 1% but no further increases in risk with ER levels beyond 5% [4]. The unadjusted ORs in our study did not increase until higher levels of ER in nonneoplastic tissue were reached. Khan *et al*. [4] also did not see as strong confounding by age as we did, although in their study the mean age of cases and controls differed by 13 years and age was significantly correlated with ER levels (r = 0.20). In an early study, Ricketts *et al. *found that mean ER levels determined by enzyme immunoassay were higher in breast cancer cases than age-matched controls [9]. Another early study found higher levels of ER in tissue taken from a quadrant opposite to a breast lesion in women having mastectomies as compared to tissue taken from reduction mammoplasties, but the ages of these groups were unknown [10]. A nested case-control study done among patients who had benign breast biopsies showing usual ductal hyperplasia found that patients who developed cancer at least six months after this time had nonsignificantly higher median expression of ER in normal tissue and a significantly higher expression of ER in the hyperplastic lesion than age-matched controls who did not develop cancer in the follow-up period [11]. A recent study showed that ER levels were nonsignificantly higher in women with fibroadenomas than women with either a strong family history without known *BRCA *mutations or a previous history of cancer suggesting that women at high risk of developing breast cancer do not have higher ER expression than women who are not at higher risk of breast cancer [12].

Differences in the methods of determining ER levels in nonneoplastic tissue likely contribute to discrepancies between this study and others, and between the sites in this study. Variation in the distribution of ER in nonneoplastic tissue is apparent among many studies [11,13-17]. We found differences in the distribution of ER levels between our study sites even when using similarly processed tissue (formalin-fixed, paraffin-embedded), using the same monoclonal antibody (6F11), having a single pathologist to score all the sections, and statistically controlling for age, case-control status and menopausal status. Other factors on which information was not available may have contributed to these differences: a delay in fixation, shorter or longer fixation time, and delay of staining prepared slides are all thought to reduce immunoreactivity with many ER antibodies [18,19]. The distribution of ER was lower in Toronto than Kingston and some degradation of the ER possibly occurred at this site. If the distribution was shifted downward similarly between cases and controls, a difference should still be seen if there was one. The difference by study site in the distribution of ER levels is a main limitation of this study; it is illustrative of differences that may have occurred between studies but have not been identified because individual studies have been conducted at a single site. If exquisite attention is paid to maintaining strict and unchanging assay methods for all samples within a study, and recording information about parameters (eg. time to fixation, fixation time) that cannot be controlled so adjustment for their effects can be made in the analysis, the observed effect may be stronger with less misclassification and it will be known more clearly if ER degradation occurs differently between cases and controls. The results from most studies are consistent with ER levels in nonneoplastic tissue being increased in women with breast cancer relative to controls.

Two interpretations of a difference in steroid hormone receptor levels in nonneoplastic breast tissue between breast cancer cases and benign breast disease controls could be that steroid hormone receptor levels increase the risk of developing breast cancer, or that steroid hormone receptor levels are influenced by adjacent tumors or other pathology. If steroid hormone receptor levels increase the risk of breast cancer, two interrelated mechanisms could be in operation. The first mechanism is that breast tissue with higher expression of ER could be more susceptible to the mitogenic effects of estrogens. Variation in the expression of ER may be due to differences in the level of differentiation of the glandular elements in the breast tissue [20], the number of intermediate cells capable of sending mitogenic paracrine signals to the epithelial cells in response to estrogens [21] and/or preneoplastic changes in the breast. Variation in the expression of ERs may have a genetic component but may also be influenced by pregnancy and lactation history. The second mechanism could involve ER levels being a marker of current estrogen levels; estrogen levels themselves could increase breast cancer risk via ER-dependent stimulation of proliferation or via DNA damage through ER-independent mechanisms by various estrogen metabolites [1,2]. Estrogen receptor levels, however, are negatively regulated by current levels of estrogens [3] and were found to be consistently related to only one marker of current estrogens in both sites in this study – age. Analyses that control for current estrogen levels measured in blood or breast tissue would help to clarify if women whose breast tissues have a tendency to express more ER in the background of a given level of estrogen would be at greater risk for developing a cancer. In this vein, Khan *et al*. 1999 [22] found that ER levels in nonneoplastic tissue were negatively associated with serum estradiol levels only among benign controls, but not in breast cancer cases. On the other hand, Bhandare *et al. *2005 [16] found that estradiol levels in ductal lavage supernatant were not associated with the ER labeling of the epithelial cells in women without cancer (but who did have a high Gail risk score for cancer), but that estrone sulphate levels were positively associated with ER labeling.

The PR gene is responsive to estrogen [3] and PR levels could be considered an indicator of the current activity of estrogens in the breast tissue – a concatenation of the levels of ER and estrogen. Few studies have examined the association between PR levels in nonneoplastic tissue and breast cancer risk. Whereas the early study by Netto *et al*. [10] found that cases had a higher distribution of PR than reduction mammoplasty controls, Khan *et al*. [4] found no significant difference between cases and controls with respect to the mean proportion of nonneoplastic cells staining for PR. We have found a reduced risk of breast cancer associated with PR levels in nonneoplastic tissue in only one study site. We had also hypothesized that women who had higher levels of PR may be at greater risk of breast cancer due to ER levels than women who had lower levels of PR because they may be the women whose breast tissues have higher exposure to estrogen. We only found a qualitative indication of this in one of our study sites; this finding, however, was not statistically significant, could be due to chance, and should be investigated in other studies. Another analysis that we performed supports this idea – the correlation between ER and PR in nonneoplastic tissue was higher in cases than controls at both sites. This suggests that for a given level of ER, the background of estrogen elicited a response, PR expression, to a greater extent in the cases than the controls. This could be due to a higher estrogen background in the cases than in the controls, or that cases have disregulated downregulation of the ER with the estrogen-dependent expression of PR.

In this study and others, steroid hormone receptors may have been different between cases and controls because they could be affected by a paracrine effect from adjacent tumors or pathologies. For example, cases could have a higher level of background estrogen around the tissue in the slides used in this study because tumor tissue is known to express estrone sulphatase, 17β-hydroxysteroid dehydrogenase, and aromatase, enzymes involved in estrogen biosynthesis [23]. Some studies have observed that the levels of aromatase and estrone sulphatase are higher in ER+/PR+ tumors than in ER-/PR- tumors [24,25], suggesting that the surrounding tissues of these tumors should differ in their ER and PR levels; this was not observed in this study but it has been in other studies [26,26-28]. The levels of enzymes involved in estrogen synthesis are also increased in benign breast conditions such as fibroadenomas [29]. Ideally, a study should be done in which steroid receptors were measured before the development of cancer.

Our study had some limitations in addition to the differences in ER levels by study site. Out of necessity, our controls were women who had a biopsy because they were suspected of harbouring a breast tumor; many had some form of benign breast changes on this biopsy but exclusion of those with a lesion associated with a much higher risk of breast cancer did not change conclusions from these results. We had a small sample size that reduced the power of subgroup analyses of interest (*eg*. by menopausal status). Steroid receptor expression and other features such as extent of differentiation and involution vary by the location of the epithelial cells (*eg*. duct versus lobule and from lobe to lobe) [14]; ER levels, however, have been shown to be quite consistent over time [30]. Measurement of other factors such as proliferation, age-related involution, ER-β, PR-A and PR-B separately, and coexpression of these factors may give a more complete picture of what makes the breast tissues of some women more susceptible to carcinogenesis than the tissues of other women.

## Conclusion

The results of this study and others are not inconsistent with a small increase in risk of breast cancer associated with ER levels in nonneoplastic tissue. Further work on the meaning of the role of ER-expressing cells in the breast combined with an attempt to control for the level of estrogens to which the cells were exposed when sampled will help to clarify this question.

## Competing interests

The authors declare that they have no competing interests.

## Authors' contributions

CGW, SKS, WMH, and KJA contributed to the study design, the preparation of the proposal for funding, interpretation of the results, and the editing of the manuscript. SKS scored the immunohistochemical staining expression in all slides. CGW conducted the data analyses and prepared the manuscript. All authors read and approved the final manuscript.

## Pre-publication history

The pre-publication history for this paper can be accessed here:


